# Assessing Lifetime Cancer Risk Associated with Population Exposure to PM-Bound PAHs and Carcinogenic Metals in Three Mid-Latitude Metropolitan Cities

**DOI:** 10.3390/toxics11080697

**Published:** 2023-08-12

**Authors:** Mohammad Aldekheel, Vahid Jalali Farahani, Constantinos Sioutas

**Affiliations:** 1 Department of Civil and Environmental Engineering, University of Southern California, Los Angeles, CA 90089, USA; aldekhee@usc.edu (M.A.); jalalifa@usc.edu (V.J.F.); 2 Department of Civil Engineering, Kuwait University, P.O. Box 5969, Safat 13060, Kuwait

**Keywords:** cancer risk, biomass burning, PAH, traffic emissions, transition metals, Los Angeles, Thessaloniki, Milan

## Abstract

Lifetime cancer risk characterization of ambient PM-bound carcinogenic metals and polycyclic aromatic hydrocarbons (PAHs) were examined in the cities of Los Angeles (USA), Thessaloniki (Greece) and Milan (Italy), which share similar Mediterranean climates but are different in their urban emission sources and governing air quality regulations. The samples in Milan and Thessaloniki were mostly dominated by biomass burning activities whereas the particles collected in Los Angeles were primary impacted by traffic emissions. We analyzed the ambient PM_2.5_ mass concentration of Cadmium (Cd), Hexavalent Chromium (Cr(VI)), Nickel (Ni), Lead (Pb), as well as 13 PAH compounds in the PM samples, collected during both cold and warm periods at each location. Pb exhibited the highest annual average concentration in all three cities, followed by Ni, As, Cr(VI), Cd and PAHs, respectively. The cancer risk assessment based on outdoor pollutants was performed based on three different scenarios, with each scenario corresponding to a different level of infiltration of outdoor pollutants into the indoor environment. Thessaloniki exhibited a high risk associated with lifetime inhalation of As, Cr(VI), and PAHs, with values in the range of (0.97–1.57) × 10^−6^, (1.80–2.91) × 10^−6^, and (0.77–1.25) × 10^−6^, respectively. The highest cancer risk values were calculated in Milan, exceeding the US EPA standard by a considerable margin, where the lifetime risk values of exposure to As, Cr(VI), and PAHs were in the range of (1.29–2.08) × 10^−6^, (6.08–9.82) × 10^−6^, and (1.10–1.77) × 10^−6^, respectively. In contrast, the estimated risks associated with PAHs and metals, except Cr(VI), in Los Angeles were extremely lower than the guideline value, even when the infiltration factor was assumed to be at peak. The lifetime cancer risk values associated with As, Cd, Ni, Pb, and PAHs in Los Angeles were in the range of (0.04–0.33) × 10^−6^. This observation highlights the impact of local air quality measures in improving the air quality and lowering the cancer risks in Los Angeles compared to the other two cities.

## 1. Introduction

Previous studies have shown that both short-term (i.e., acute) and long-term (i.e., chronic) exposure to fine particulate matter (PM_2.5_) can lead to increased cardiopulmonary morbidity and mortality in humans [1,2]. According to the International Agency for Research in Cancer (IARC), an agency of the World Health Organization (WHO), the second most common cancer disease for all ages and both sexes is lung cancer with approximately 2.21 million reported cases worldwide in 2020 [3]. It was estimated that prolonged exposure to ambient PM_2.5_ could lead to approximately 5% of bronchus, trachea, and lung cancer mortality in urban areas around the world [4]. Various studies have attributed lung cancer to the inhalation of carcinogenic species of ambient PM, including polycyclic aromatic hydrocarbons (PAHs) and redox active metals (e.g., arsenic, cadmium) [5,6,7,8].

PAHs are a broad group of chemical compounds composed of multiple fused aromatic rings of carbon and hydrogen atoms that can be arranged in a linear, angular, or clustered configuration with varying complexity and lipophilic properties [9,10]. Particulate phase PAHs are low-volatility toxic organic compounds that have the potential to travel long distances, thus developing genotoxic effects when inhaled by humans [11,12]. They can originate from a wide range of sources, including road traffic (i.e., automobile engines), incomplete combustion of fuels in industrial activities, cooking, and biomass burning [10,13,14]. The United States Environmental Protection Agency (USEPA) has identified numerous PAH species as priority pollutants due to their potential to cause mutagenesis and carcinogenesis [15,16]. In particular, benzo(α)pyrene (BaP) has been widely employed in cancer risk assessment studies as a surrogate for all PAHs due to its established and potent carcinogenic properties [17,18]. This approach involves converting the concentrations of all targeted PAHs to BaP-equivalent concentrations using potency equivalent factors (PEFs) [19]. Moreover, several toxic metal species present in ambient PM which, when inhaled, can cause serious health deterioration and carcinogenic effects in humans, including nose, liver, kidney, and lung cancers [6,20]. According to IARC, chromium VI (Cr(VI)), arsenic (As), cadmium (Cd), and metallic nickel (Ni) have all been classified as Group 1 carcinogens, indicating sufficient and strong evidence of their ability to cause cancer in humans [21]. Research has found that vehicular and industrial emissions are the primary contributors to high levels of heavy toxic metals in ambient PM in various developed and developing countries [22,23].

The three cities Investigated In this study, namely Los Angeles, Milan, and Thessaloniki, are densely populated urban centers and are vulnerable to various carcinogenic pollutants emitted from a multitude of sources, posing a significant risk to the health of their inhabitants [24]. Previous source apportionment studies in Los Angeles have demonstrated various urban emission sources contributing to the formation of fine particulate pollutants in the region [25,26,27]. Hasheminassab et al. (2014) [25] found that the Los Angeles basin was primarily affected by vehicular traffic-related sources, as well as secondary ammonium nitrate and sulfate formation. However, since 2007, state and federal regulations have been implemented to limit traffic-related pollution, resulting in significant reductions in vehicular emissions. In Thessaloniki, ambient PM pollution was mainly caused by vehicular emissions and residential heating [28,29]. Argyropoulos et al. (2016) [30] concluded that burning biomass had a much greater effect on PM redox activity in the colder months due to the rise in residential wood burning during the winter following the economic crisis in Greece in 2009. Exposure to PM emissions related to wood burning has been linked to various adverse health impacts due to the presence of redox active species, including PAHs [30,31,32]. In Milan, the major emission sources during the summer season were traffic and secondary organic aerosol (SOA), while intense biomass burning was the major source in the winter period [33,34]. During the wintertime in the metropolitan area of Milan, research found that ambient PM caused premature cell division and DNA damage, which was linked to increased concentrations of PAHs and transition metals [35]. Hakimzadeh et al. (2020) [34] reported that burning of biomass in combination with atmospheric stability during the winter months led to substantially elevated levels of PM_2.5_ oxidative potential in the region, surpassing the values recorded in numerous European cities (e.g., Thessaloniki) and even in Los Angeles.

Analyzing the long-term trend in ambient PM levels, Los Angeles, Thessaloniki, and Milan have demonstrated varying degrees of success in reducing their PM_2.5_ concentrations over recent decades. According to the South Coast Air Quality Management District (AQMD), a governmental agency in southern California, the annual-averaged PM_2.5_ concentration in Los Angeles has decreased from 28.5 ± 8.5 µg/m^3^ in 2005 to 11.8 ± 4.2 µg/m^3^ in 2022, which still remains slightly higher than the recommended annual mean PM_2.5_ concentration of 10 µg/m^3^ set by the World Health Organization [36]. In Thessaloniki, the annual ambient PM_2.5_ concentration has also significantly decreased from 97 ± 18.5 µg/m^3^ in 1994–1995 [37] to 15.7 ± 5.5 µg/m^3^ in 2019–2021 [38]; however, the concentrations are still higher than Los Angeles and the WHO annual mean PM_2.5_ level. Considering Milan, the long-term ambient PM_2.5_ concentrations exhibited a slight decrease from 54.5 ± 11.6 µg/m^3^ in 1997–1998 [39] to 44.22 ± 12.81 µg/m^3^ in 2018–2019 [34], approximately four times higher than Los Angeles and the WHO annual mean PM_2.5_ concentration (10 µg/m^3^).

This study aimed to assess the potential lifetime cancer risk linked to PM-related carcinogenic metals and PAHs in three metropolitan areas, including Los Angeles, Milan, and Thessaloniki. These cities share the same mild Mediterranean climate; however, they are characterized by distinct pollution sources and air quality regulations. Therefore, the main purpose of this work was to investigate changes in PM concentrations and the associated cancer risk due to the variability in emission sources and their intensities in the three analyzed regions. Given the great variability of indoor concentrations and sources of pollutants, our focus is primarily on the health risks associated with exposure to outdoor pollutants. The results of this work can be used as a potential guide for researchers and medical professionals to evaluate the impact of exposure to ambient carcinogenic components and assist government officials to make more informed decisions regarding the adoption of air quality policies.

## 2. Materials and Methods

### 2.1. Description of Sites

Los Angeles, Milan, and Thessaloniki are densely populated urban centers and particularly vulnerable to various carcinogenic pollutants emitted from a multitude of sources [24]. The topography of Los Angeles, which is surrounded by mountains on three sides and faces the Pacific Ocean to the west, along with temperature inversions can result in the accumulation of pollutants, especially in the eastern region of the basin due to the typical direction of the sea breeze coming from the west [40]. Moreover, Milan is located in Po valley, among the most industrialized regions in northern Italy, and has been identified as one of the most contaminated regions in Western Europe [41]. The city is densely populated, accommodates a significant number of vehicles and motorcycles, and has been impacted by various urban emission sources. Additionally, the topography of the region (i.e., the presence of the Alps mountains in the north) coupled with the meteorological conditions, especially in the winter season, further contributed to the deterioration of air quality by limiting the vertical and horizontal dispersion of PM emitted in the valley [42]. Furthermore, Thessaloniki, the second most populous city in Greece, has a population of more than a million people and is widely regarded as one of the most polluted cities in Europe in terms of air quality [43]. The region has a Mediterranean climate, with an average temperature of 7 °C in winter and 25 °C in summer, and is surrounded by mountains in the east and northeast (i.e., mount Chortiatis), Thermaikos Gulf in the south, and flat terrain in the west side.

**Figure 1:**
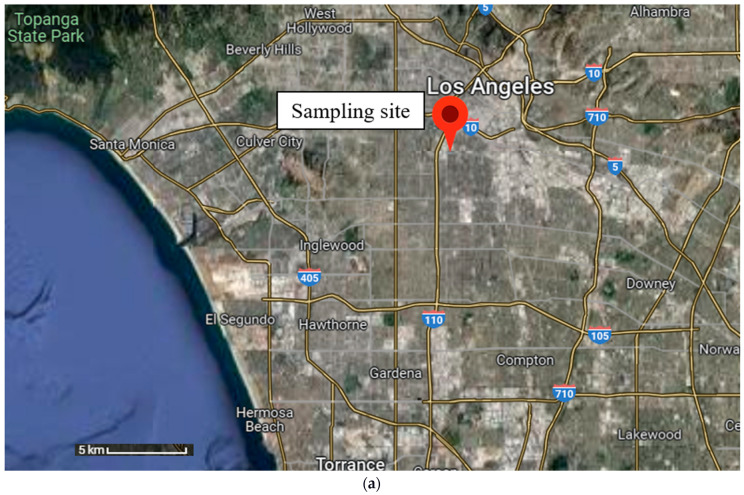

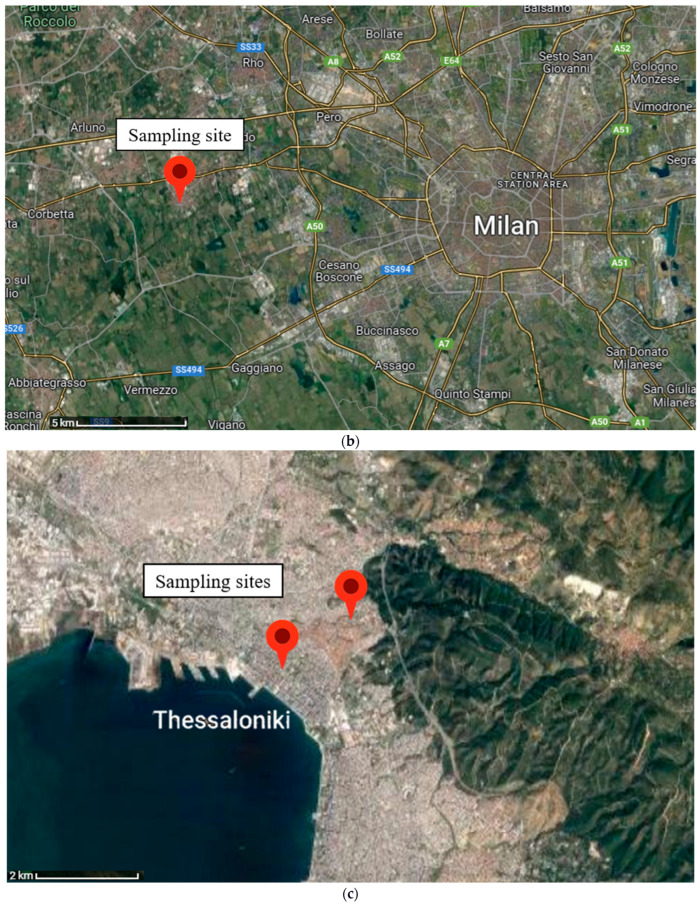
Geographical maps of the sampling sites in (**a**) Los Angeles, (**b**) Milan, and (**c**) Thessaloniki. (Source: Google Maps).

Figure 1 shows the geographical location of the sampling sites in the three metropolitan cities. The sampling sites selected in Milan, Thessaloniki, and Los Angeles represent specific environments characterized by local sources of emissions and climatic conditions. These specific sites were utilized in previous research studies conducted in the three metropolitan areas. In Milan, Mousavi et al. (2019) [44] and Altuwayjiri et al. (2021) [42] conducted their sampling in a suburban residential area of Bareggio, located about 10 km to the west of the city center. This specific location is characterized by significant vehicular and residential emissions and has been previously selected as an urban background for assessing public exposure to the baseline levels of ambient PM [34,42,44]. The predominant PM emission sources in this site were traffic (i.e., vehicular exhaust and resuspended road dust) and secondary organic aerosol (SOA) during the summer season and intense biomass burning (i.e., residential heating) during the winter season [34]. In Thessaloniki, the sampling was conducted in two distinct locations, including the urban background (UB) and the urban traffic (UT), which were heavily used in previous research studies [45,46,47,48]. The urban background site was located in a residential area in the northern part of Thessaloniki on the roof of a monitoring station (5 m from the ground), where samples were mostly dominated by biomass burning emissions due to elevated residential heating activities. Furthermore, this site was also influenced by mild traffic emissions as well as secondary PM formation [28]. On the other hand, the urban traffic site was situated in a commercial city center located near one of the heavily congested roadways in downtown Thessaloniki. This site was predominantly impacted by anthropogenic sources, including vehicular exhaust, tire wear, brake abrasion, as well as biomass burning [30]. In Los Angeles, the sampling was carried out in the particle instrumentation unit (PIU) at the University of Southern California, which is situated in close proximity to a major freeway (i.e., I-110). This location has often been used in prior studies as it offers a combination of various urban pollutant sources, releasing PM in a variety of sizes and chemical constitutions [49,50,51,52]. The samples in this region were mostly impacted by vehicular exhaust particles transported from the freeway by the dominant southwesterly winds [27]. Additionally, this location was influenced by soil and road dust, urban background, and secondary aerosols [27,53].

### 2.2. Sampling Information

Three sets of PM_2.5_ samples, each collected at a unique location site, including Los Angeles, Milan, and Thessaloniki, were employed in this study. A summary of sampling information for each of these PM batches is shown in Table 1. In Los Angeles, PM_2.5_ samples, each representing 2–3 days of sampling, were collected during two periods of summertime (August 2018) and wintertime (December 2018 to January 2019). The field campaign in Milan was conducted from December 2018 to February 2019 and from May 2019 to July 2019 to collect weekly PM_2.5_ samples. The samples investigated in Thessaloniki consisted of two main campaigns: (1) daily (i.e., 24 h) PM_2.5_ sampling from February 2012 to March 2012 and from January 2013 to February 2013; (2) 48 h PM_0.49_ (particles with aerodynamic diameter ≤ 0.49 µm) sampling from January 2013 to March 2013 and from May 2013 to June 2013.

In Los Angeles, the Versatile Aerosol Concentration Enrichment System (VACES) was utilized to collect PM_2.5_ samples [27]. The sampler drew ambient aerosols at 300 lpm and then the flow was saturated with water vapor and split into three parallel lines, each at 100 lpm. The particles were grown into droplets after passing through a cooling tube where the air temperature was reduced. Virtual impactors were employed in the sampling lines to enrich the concentration of the particles by 20-fold. After this stage, diffusion dryers filled with silica gel were used to remove excess moisture of the particles, which were then collected on 37 mm diameter quartz filters. In Milan, the Personal Cascade Impactor Sampler (PCIS), operating at a flow rate of 9 L/min, was employed to collect ambient PM_2.5_ samples on prebaked quartz filters [34]. To sample PM_2.5,_ the first impaction stage (cut-point of 2.5 µm) was used to remove coarse PM (particles with aerodynamic diameter > 2.5 µm). The weekly average PM_2.5_ collected mass was measured gravimetrically, by determining the difference between the unloaded and loaded quartz filters. In Thessaloniki, two types of samplers were employed. The first was a low-volume impactor, operating at a constant flow rate of 2.3 m^3^/h [28]. Ambient PM_2.5_ were collected on both 47 mm Teflon filters as well as prebaked 47 mm quartz filters. The second sampler was a high-volume cascade impactor, used to collect PM_0.49_ samples during both the cold and warm periods of the year [30]. This sampler was operated at a constant flow rate of 1.1 m^3^/min with PM_0.49_ collected on prebaked quartz filters.

### 2.3. Chemical Analysis

PM samples were analyzed for metal elements using inductively coupled plasma mass spectroscopy (ICP-MS). First, a section of each filter was initially solubilized in an acid mixture containing nitric acid, hydrochloric acid, and hydrofluoric acid (0.6 mL 16N HNO3, 0.2 mL 12 N HCl, 0.1 mL 28N HF) by employing an automated microwave-assisted digestion system (Milestone ETHOS+). The samples were then analyzed using ICP-MS, with standard plasma conditions for most elements and cool plasma/shielded torch conditions for lighter elements. This methodology, coupling microwave-assisted acid digestion with ICP-MS, proved to be an accurate and sensitive method for analyzing a broad range of elements in PM loaded samples [54]. In order to quantify the mass concentration of particle-phase PAHs, a portion of each filter was subject to ultrasonic extraction using a dichloromethane (DCM)/n-hexane mixture. A glass column filled with alumina and silica gel was employed in order to separate PAHs and remove any other unwanted species from the extract. PAH analysis was performed using a gas chromatograph integrated with a mass spectrometric detector (GC/MS). PAHs were identified through the application of the standard PAH mixture and by comparing the fragmentation patterns of compounds in the sample to those present in the National Institute of Standards and Technology (NIST) mass spectral library. More details related to the analysis of PAHs can be found in Chrysikou et al. [55]. It should be noted that all concentrations of PM chemical species were blank corrected.

### 2.4. Health Risk Characterization

#### 2.4.1. Carcinogenic Metals

The cancer risk associated with inhalation exposure to metal elements was quantified by employing inhalation unit risks (IUR) for each element. The IUR is defined as the upper-bound excess lifetime cancer risk that may be incurred from continuous exposure per 1 µg/m^3^ of a component’s ambient concentration [56]. This approach has been extensively employed to assess exposure risk via inhalation [57,58,59]. In this method, the concentration of individual metals is multiplied by their corresponding IUR values to obtain the lifetime cancer risk values. However, it is important to incorporate the different indoor and outdoor exposure times as well as the difference in indoor and outdoor pollutant levels in the calculation of the lifetime cancer risk. By assuming that people will spend 80% of their time indoors and therefore, 20% outdoors [5], the lifetime individual cancer risk can be estimated using the following equation:

(1) $$LICR=IUR\times \left(0.2\times C_{i_{outdoor}}+0.8\times C_{i_{indoor}}\right)$$

where LICR is a dimensionless unit indicating the lifetime cancer risk through inhalation of carcinogenic metals; IUR shows the inhalation unit risk (per $\mu g/m^{3}$) reported by the Integrated Risk Information System (IRIS) based on the epidemiological lifetime exposure (Table 2); $C_{i_{outdoor}}$ and $C_{i_{indoor}}$ are the concentration of the metal i in outdoor and indoor environments, respectively ($\mu g/m^{3}$). The outdoor concentration values correspond to the average values extracted from the PM samples. Since the indoor emission sources and their relative contribution is subject to extreme variations depending on the buildings’ conditions and residents’ activities, including a variable corresponding to indoor pollutant sources will impose great uncertainty in our calculation. Thus, the risk assessment in this study was solely focused on exposure to outdoor emission sources, making infiltration of outdoor pollutants the sole indoor source in our investigation. The indoor concentration of a PM component ($C_{i_{indoor}}$) is therefore obtained by multiplying the outdoor concentration ($C_{i_{outdoor}}$) by the infiltration factor ($F_{inf}$), which represents the portion of ambient PM that penetrates the indoor space and remains suspended in the air. However, we should note that this approach simplifies the governing processes in infiltration and deposition of the indoor particles as the impacts of air exchange rate and particle size on the deposition and accumulation of the particles, which could increase their indoor concentration, have not been considered. The infiltration factor used in this study is discussed in Section 2.4.2.

#### 2.4.2. Polycyclic Aromatic Hydrocarbons (PAHs)

The lifetime lung cancer risk linked to exposure to PM-bound PAHs was estimated using the BaP equivalent method (${BaP}_{eq}$). This approach has been adopted in several studies to assess the carcinogenic risks of airborne PAHs [5,59,60,61]. Benzo(a)pyrene (BaP), which is considered the most potent PAH compound, is used as a proxy for the PAH fraction of complex mixtures. The carcinogenic potency of other PAH compounds is determined relative to that of BaP. The concentration of ${BaP}_{eq}$ for each PAH component is quantified by multiplying the concentration of individual PAHs by their corresponding potency equivalent factor (PEF). The total ${BaP}_{eq}$ is then estimated by adding the individual ${BaP}_{eq}$ values, as shown in the following equation:

(2) $${BaP}_{eq}=\sum_{i=1}C_{i}{\;\times \;PEF}_{i}$$

where $C_{i}$ is the concentration of the PAH compound i and ${PEF}_{i}$ is the corresponding potency equivalent factor (PEF). The Office of Environmental Health Hazard Assessment (OEHHA) reported a PEF value of 1 for Benzo(a)pyrene and Dibenzo(a)pyrene; 0.1 for Benzo(b)fluoranthene, Benzo(k)fluoranthene, Benzo(j)fluoranthene Benz(a)anthracene, and Indeno(1,2,3-cd)pyrene; 0.01 for Anthracene, Chrysene, Benzo(g,h,i)perylene; and 0.01 for Phenanthrene, Fluoranthene, Acephenanthrylene, and Pyrene [62]. The lifetime lung cancer risk from exposure to PAHs is then calculated using Equation (1) and substituting $C_{i}$ with total ${BaP}_{eq}$ values. The IUR value corresponding to BaP is 0.0006 per µg/m^3^.

In our model, we considered three distinct scenarios to approximate real-world variations in indoor pollutant infiltration. The Worst-case Scenario (WS) assumed peak infiltration of particles into the indoor environment, typically due to open windows, doors, and poor indoor ventilation. The Best-case Scenario (BS) assumed limited infiltration as a result of closed windows and doors, constraining the penetration of outdoor pollutants. Finally, the Mixed Scenario (MS) represented a mixture of the two previous scenarios and is closer to real-world conditions. We assumed the infiltration factor value of 0.8 and 0.4 for WS and BS, respectively, which is within the range of the infiltration factors reported during the opened window and closed window conditions in the literature [63,64,65,66,67]. The infiltration factor associated with the mixed scenario was assumed to be 0.6, which is the average of BS and WS infiltration factors. Although the WS, BS, and MS scenarios provide a reasonable estimate of the lifetime cancer risk, it is important to note that these estimates are based on current conditions and assume constant concentrations of PM species over a lifetime. In reality, future changes in these concentrations could impact these estimates. These changes could be due to numerous factors such as improvements in emission control technologies, changes in regulations, shifts in population and traffic densities, and advancements in indoor air purification systems. Therefore, the actual lifetime cancer risk could be lower or higher than the estimates provided in this study, depending on how these factors evolve over time.

## 3. Results and Discussion

### 3.1. Concentration of Carcinogenic Metals and PAHs

**Figure 2:**
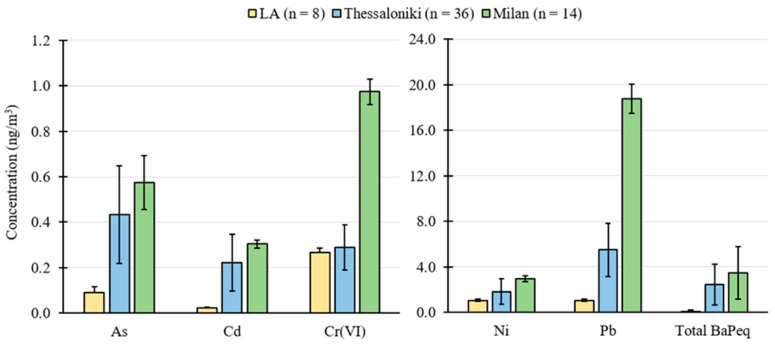
Comparison of ambient concentration of carcinogenic metals and total ${BaP}_{eq}$ in Los Angeles (LA), Thessaloniki, and Milan. The *y*-axis represents the average mass concentration of the summer and winter seasons. The term “n” refers to the number of samples.

Figure 2 shows the average concentration of carcinogenic metals extracted from samples in Los Angeles, Milan, and Thessaloniki. The redox active metals investigated in this study for their carcinogenic impact on humans were Arsenic (As), Cadmium (Cd), Hexavalent Chromium (Cr(VI)), Nickel (Ni), and Lead (Pb), which all have been identified as potentially carcinogenic through an inhalation pathway [68,69]. Chromium is present in the atmosphere in mainly two valence states of non-carcinogenic Cr(III) and carcinogenic Cr(VI). The concentration of Cr(VI) was estimated according to the reported ratio of carcinogenic Cr(VI) to total Cr concentration in the literature (i.e., 1/7) [59,70,71,72]. Pb and Ni demonstrated the highest loadings on the collected samples, followed by trace levels of Cr(VI), As, and Cd, with concentrations below 1 ng/m^3^. The results revealed a strong variation in the levels of carcinogenic metals across the studied metropolitan areas. The concentrations of As and Cd in the samples collected in Los Angeles differed significantly from those of Milan and Thessaloniki, as did the concentrations of Pb and Ni. The significant difference in the concentration of heavy metals in Los Angeles can be attributed to the impact of after-treatment and local air quality policies in reducing the contribution of combustion sources. Additionally, similar levels of Cr(VI) are observed in both Los Angeles and Thessaloniki given that it can be released from a multitude of sources, as discussed in Section 3.2. Table 3 summarizes the average concentration of PAH components measured at the investigated location sites. In general, the proportions of outdoor 3-ring PAHs were the lowest among all location sites compared to heavy molecular weight PAHs (i.e., compounds of four or more aromatic rings). Among the three studied urban areas, the highest proportions of PM-bound PAHs with three rings were found in Thessaloniki samples. The total concentration of PAH components in this region was 11.30 ± 9.43 ng/m^3,^ with benzo(g,h,i)perylene and benzo(e)pyrene showing the highest levels. Furthermore, Milan exhibited the highest levels of particle-bound PAHs, with a total concentration of 31.83 ± 28.68 ng/m^3^. Ambient levels of chrysene and several high molecular weight PAHs (e.g., benzo(b)fluoranthene, benzo(k)fluoranthene, benzo(e)pyrene) were considerably elevated during the sampling periods in this urban site. This is largely the result of significantly higher PAH emissions during the wintertime due to increased biomass combustion, raising the annual average values [34]. In fact, the total PAH concentration in Milan was not only higher than our measurements in Thessaloniki and Los Angeles, but also exceeded the measurements in most cities, including Florence [73], Zaragoza, and Monzon [74]. On the contrary, we observed low levels of PAHs in Los Angeles, with multiple components registering values below the detection limit. The total concentration of PAHs in Los Angeles was 0.88 ± 0.50 ng/m^3^.

It should be highlighted that the sampling period in Thessaloniki was not the same as Los Angeles and Milan. However, we should stress that the samples collected in these three metropolitan cities can be compared effectively with each other. Despite the fact that the samples from Thessaloniki were collected in 2013, five years prior to the LA and Milan samples (2018–2019), they remain pertinent for inclusion in our study. This is due to the observation that ambient concentrations of PM constituents (i.e., PAHs, carcinogenic metals) in Thessaloniki did not show substantial changes in the recent decade. This is supported by Figure S1, which shows the PAH and carcinogenic metal concentrations in Thessaloniki from more recent sampling campaigns conducted by Karageorgou et al. (2021) [75] and Besis et al. (2023) [76] in 2015–2018 and 2020, respectively. As shown in the figure, these concentrations align with the levels reported by Saffari et al. (2013) [28] and Argyropoulos et al. (2016) [30] that we utilized in our study, thereby validating the comparison of samples from different sampling periods.

### 3.2. Primary Emission Sources of Carcinogenic Metals and PAHs and Mitigation Strategies

The primary sources of carcinogenic metals (i.e., As, Cd, Cr(VI), Ni, and Pb) in the metropolitan areas of Los Angeles, Milan, and Thessaloniki are associated with specific traffic, industrial, and combustion activities. Table 4 provides a summary of all possible primary sources of carcinogenic metals and PAHs. In particular, the emissions of As, Cd, and Ni are predominantly linked to industrial processes, specifically metallurgical activities, as well as oil combustion [77,78]. Additionally, sources of lead (Pb) are primarily linked to industrial activities, including the manufacturing of lead–acid batteries, iron and steel industries, and the production of ceramics [79,80]. In Los Angeles, Pb primarily originates from non-tailpipe emissions, whereas Ni is mainly emitted by industrial activities (e.g., stainless steel manufacturing) as well as refineries and power plants due to fuel combustion [81,82]. On the other hand, Pb in Milan is predominantly derived from road dust and industrial activities (e.g., crystal and glass manufacturing) [83], while Ni comes mainly from vehicle exhausts and oil combustion [33]. Pb emissions in Thessaloniki are directly linked to vehicle exhausts, road dust, and biomass burning [84], while Ni is associated with fossil fuel combustion in shipping and industrial processes [85]. PM-bound Cr is typically emitted from metal processing, coal burning, and fossil fuel emissions. Vehicular emissions can also increase the concentration of airborne Cr in urban areas with heavy diesel traffic [86,87]. Previous studies in Los Angeles have linked Cr (especially (Cr VI)) emissions with industrial facilities, including metal processing facilities [82]. However, the ambient Cr levels in Thessaloniki can be attributed to oil combustion and vehicular diesel emissions [37,85,88]. Furthermore, PAHs are emitted from incomplete fuel combustion processes in vehicle exhausts and various industrial stacks (e.g., heavy oil plant, power plant, and cement plant, waste incinerators, commercial kitchens) as well as residential combustion (e.g., heating, cooking) [5,89,90,91,92]. The enhanced PAH levels during the cold season in Milan and Thessaloniki are mostly driven by biomass combustion for residential heating [28]. This observation is corroborated by high levels of chrysene, benz(a)anthracene, and benzo(a)pyrene in Table 3, which are tracers of biomass burning [29].

Considering the health hazards linked to carcinogenic pollutants, it is imperative to develop effective mitigation strategies and rigorous environmental policies aimed at minimizing the associated health risks. In the United States, both federal and state governments have developed standards to regulate various air pollutants and control their sources [26]. USEPA established the National Ambient Air Quality Standards (NAAQS) in 1987 in order to regulate ambient concentrations of criteria pollutants (e.g., PM_2.5_, PM_10_) [93,94]. In the state of California, policymakers and air quality agencies have implemented stricter plans to regulate emission sources, especially motor vehicles, and attain PM_2.5_ standards [25]. The California Air Resources Board (CARB) has promulgated stringent low-emission vehicle (LEV) regulations to reduce tailpipe emissions, including criteria pollutants and greenhouse gases from medium- and light-duty vehicles [95]. According to a study examining the long-term trend of air pollutant sources in Los Angeles, the contribution of combustion emissions to the ambient concentration of PM_2.5_-bound metals decreased by nearly 88% during the 2005–2018 period due to the effective emission control policies in the region [96]. The long-term reduction in PM concentrations in Los Angeles could minimize the associated carcinogenic risk since there is an established correlation between airborne PM exposure and lung cancer mortality [1]. On the other hand, Milan and Thessaloniki are impacted by various pollution sources, including vehicular exhaust, industrial operations, and particularly biomass burning during the cold season which increases PAH levels and exacerbates health complications. Therefore, these two cities could greatly benefit from implementing stricter air quality standards and advocating for efficient after-treatment processes, similar to those implemented in Los Angeles, in order to yield significant reductions in the concentration of carcinogenic pollutants. In addition, the transition to cleaner alternatives in transportation and industry, such as the adoption of renewable energy sources and electric vehicles, could also mitigate pollutant emissions. For reduction in PAH emissions, promoting efficient combustion practices, particularly in residential heating and cooking, coupled with transitioning to cleaner fuels, can have a substantial impact as well.

### 3.3. Cancer Risk Assessment

Table 5 compares the total ${BaP}_{eq}$ in the investigated location sites as well as the reported values in the literature. Our results indicated that Milan’s enhanced PAH concentration translated into the highest total ${BaP}_{eq}$ value among the urban sites examined in this study. The total ${BaP}_{eq}$ in Milan was 1.42 times higher than that of Thessaloniki, which is impacted by similar biogenic and anthropogenic sources. However, the estimated ${BaP}_{eq}$ in LA was approximately 25 and 35 times lower than the corresponding values in Milan and Thessaloniki, respectively. It is important to highlight the impact of biomass burning emissions in both European cities (i.e., Milan and Thessaloniki), which significantly increased the PAH levels and, consequently, the total ${BaP}_{eq}$ values. In fact, the ${BaP}_{eq}$ value in Milan was higher compared to observed values in China and other European location sites. Additionally, we should note that Kam et al. (2013) [97] conducted PM sampling inside the I-110 freeway; however, Pirhadi et al. (2020) [27] carried out the sampling in an urban area. This distinction in sampling locations can clarify the reduction in the total ${BaP}_{eq}$ level observed in Pirhadi et al. (2020) [27] study.

Table 6 shows the lifetime cancer risk values according to the concentration of metal elements and ${BaP}_{eq}$ values obtained in Los Angeles, Thessaloniki, and Milan. The total risk value represents the cumulative risk factor from exposure to the investigated metal elements and PAHs. We should note that all investigated species were collected in one shared filter simultaneously during each day of sampling. The table presents three PM infiltration scenarios for each location site as discussed previously in the methodology section. According to the table, Cr(VI) poses the highest lifetime critical risk among the studied PM components, with LICR values exceeding the minimal acceptable risk level by a considerable margin among all location sites and scenarios. The margin was most pronounced in Milan, where the lifetime cancer risk was 5–10 times higher compared to the US EPA standard value (10^−6^) [100]. The high carcinogenic risk of Cr(VI) stems from its ability to interact with critical cell components (e.g., DNA and nuclear proteins), causing DNA damage, oxidative stress, and possible carcinogenesis [101]. As discussed earlier, Cr(VI) is predominantly produced from a wide range of anthropogenic sources, including industrial activities, combustion processes, and vehicular exhausts [102,103]. The cancer risk values of Ni, Pb, and Cd in all studied cities and across different scenarios were considerably lower than EPA’s standard value, with LICR values in the range of 0.01–0.46. Moreover, Arsenic registered LICR values higher than the US EPA standard in both Milan and Thessaloniki. In general, we observed a similar pattern in both cities, where the risk values corresponding to As, PAHs, and Cr(VI) were higher than the standard level in most scenarios. However, the cancer risk values in Los Angeles were noticeably lower compared to the other location sites. Most remarkably, the risk from PAH exposure did not exceed the acceptable value even in the worst-case scenario (i.e., highest infiltration rate) in this region. As mentioned earlier, our sampling site in LA is located in the heart of the urban section of the city and near a major vehicle roadway, denoting traffic and residential heating emissions as the potential drivers of the concertation of PAHs in this city. However, the possible impact of residential/biomass burning emissions is curtailed by the short cold periods, turning traffic emissions into the prime source of PAH components at this sampling site. Therefore, the low cancer risk associated with PAH levels highlights the impact of air quality policies targeting combustion emissions in reducing the release of carcinogenic components in LA. This is further supported by the cancer risk estimation of other PM components which were mostly below the EPA standard. It is important, however, to note that the collective cancer risk values from all carcinogenic components and PAHs exceeded the EPA standard across all location sites. For instance, the corresponding value even in Los Angeles was above 10^−6^ even for the scenario with the lowest infiltration of outdoor pollutants into indoor environment (i.e., 2.07 × 10^−6^). As expected, Milan showed the highest total cancer risk, reaching almost 15 times the EPA standard.

## 4. Summary and Conclusions

This study investigated the lifetime cancer risk from population exposure to carcinogenic PM-bound components in three urban environments including Los Angeles, Milan, and Thessaloniki which shared similar Mediterranean climates but were different in their emission sources and governing air quality policies. According to our results, Milan exhibited the highest lifetime cancer risk values compared to the corresponding values in Los Angeles and Thessaloniki, with values ranging from (0.19 ± 0.02) × 10^−6^ to (9.82 ± 0.58) × 10^−6^. The population exposure to As, Cr(VI), and PAHs in Milan exceeded the US EPA standard across all scenarios, highlighting the magnitude of the air pollution in this metropolitan area. We also observed lower concentrations of heavy metals and PAHs in Los Angeles. The PAH levels were significantly reduced compared to our measurements in this basin, six years prior. The lower mass concentration of particle-bound components in Los Angeles translated into the lowest cancer risk estimations among the examined sites. However, the cancer risk from exposure to a highly carcinogenic metal (i.e.,Cr(VI)) was still above the US EPA’s acceptable risk levels. Similar to Milan and Thessaloniki, the cumulative cancer risk value corresponding to exposure to carcinogenic metals and PAHs in Los Angeles exceeded the acceptable levels, highlighting the need for additional measures to target the local emission source in this region. Furthermore, it is essential to highlight that other environmental and non-environmental factors could contribute to the prevalence of lung cancer in the analyzed cities. Our study focused only on the exposure to main carcinogenic PM-bound species, which was a part of other factors impacting public health outcomes, including tobacco smoking, electronic cigarettes, exposure to additional pollutants (e.g., radon), lifestyle factors (e.g., poor diet), and inherited genetics [104,105]. Therefore, future research is needed to integrate our results with this complex array of factors to further understand the interplay between environmental factors, urban living conditions, and public health outcomes.

Our study, although comprehensive, has certain limitations and uncertainties which need to be highlighted. Firstly, the data collected do not cover all seasonal variations as the sampling periods only included the summer and winter seasons. Secondly, different sampling techniques were used in each city, which may affect the direct comparability of data across the studied locations. Additionally, a major source of uncertainty lies in the estimation of indoor concentrations using varying infiltration factors. In our model, we considered three scenarios: Worst-case Scenario, Best-case Scenario, and a Mixed Scenario to model variations in indoor pollutant infiltration due to changes in ventilation conditions. Even though these scenarios cover a range of possible conditions, there is still inherent uncertainty due to the variation in building designs, ventilation systems, seasonal meteorology, and lifestyle factors that influence indoor pollutant levels. For example, using ventilation systems equipped with in-line filters can help to reduce indoor pollutant levels and consequently minimize the cancer risk associated with indoor exposure. On the other hand, personal habits such as indoor smoking or frequent use of chemical cleaning products can increase indoor pollutant concentrations, potentially raising the associated cancer risk. Furthermore, the assumptions made under our cancer risk model introduced potential uncertainty. For instance, the model assumed that individuals spend 80% of their time indoors and 20% outdoors. This approximation might not be representative of all individual lifestyles, potentially affecting the accuracy of our risk estimates. Our model also considers health risks associated with exposure to outdoor pollutants only due to the great variability of indoor concentrations and sources of pollutants.

## Figures and Tables

**Table 1:** Summary of information pertaining to the collected particles in Los Angeles, Milan, and Thessaloniki.

City	Particle Size	Sampler	Flow Rate	Number of Samples	Filter Type	Sampling Period(s)	Study
Los Angeles, USA	PM_2.5_	Versatile Aerosol Concentration Enrichment System (VACES)	300 L/min	8	Quartz	August 2018 December 2018–January 2019	Pirhadi et al., 2020 [27]
Milan, Italy	PM_2.5_	Personal cascade impactor sampler (PCIS)	9 L/min	14	Quartz	December 2018–February 2019 May–July 2019	Hakimzadeh et al., 2020 [34]
Thessaloniki, Greece	PM_2.5_ and PM_0.49_	Low-volume impactor with 2.5µm cutpoint High-volume impactor with 0.49µm cutpoint	38 L/min 1100 L/min	26 10	Quartz and Teflon Quartz	February–March 2012 January–February 2013 January–March 2013 May–June 2013	Saffari et al., 2013 [28] Argyropoulos et al., 2016 [30]

**Table 2:** Summary of information pertaining to the collected particles in Los Angeles, Milan, and Thessaloniki.

Metal	As	Cd	Cr(VI)	Ni	Pb
IUR value [56] (per $\mu g/m^{3}$)	0.0043	0.0018	0.012	0.00024	0.000012

**Table 3:** Concentration of particle phase PAHs (ng/m^3^) in Los Angeles, Thessaloniki, and Milan. The values below detection limit are indicated as BDL. The term “n” refers to the number of samples.

PAH Species	Los Angeles (n = 8)	Thessaloniki (n = 36)	Milan (n = 14)
Phenanthrene	0.04 ± 0.02	0.425 ± 0.33	0.307 ± 0.191
Retene	BDL	BDL	0.383 ± 0.291
Anthracene	BDL	0.165 ± 0.121	BDL
Pyrene	0.032 ± 0.012	0.794 ± 0.546	1.035 ± 0.638
Chrysene	0.023 ± 0.017	1.212 ± 0.932	5.228 ± 3.399
Benz(a)anthracene	0.014 ± 0.011	1.025 ± 0.847	1.458 ± 0.934
Acephenanthrylene	BDL	0.136 ± 0.051	0.096 ± 0.091
Fluoranthene	0.082 ± 0.031	0.774 ± 0.566	1.108 ± 0.689
Benzo(ghi)fluoranthene	0.012 ± 0.009	BDL	2.2 ± 1.401
Benzo(b)fluoranthene	0.071 ± 0.048	0.586 ± 0.394	5.613 ± 3.514
Benzo(k)fluoranthene	0.108 ± 0.042	0.951 ± 0.911	5.159 ± 3.279
Benzo(e)pyrene	0.081 ± 0.044	1.316 ± 0.957	3.896 ± 2.461
Benzo(a)pyrene	0.071 ± 0.047	1.128 ± 0.754	0.155 ± 0.13
Benzo(g,h,i)perylene	0.201 ± 0.099	1.476 ± 1.139	1.791 ± 1.086
1-Methylchrysene	BDL	BDL	0.497 ± 0.318
Perylene	BDL	BDL	BDL
Benzo(j)fluoranthene	BDL	BDL	0.192 ± 0.132
Dibenz(a,h)anthracene	BDL	0.188 ± 0.141	0.37 ± 0.239
Picene	BDL	BDL	0.233 ± 0.146
Cyclopenta(cd)pyrene	BDL	BDL	BDL
Indeno(1,2,3-cd)pyrene	0.104 ± 0.044	1.128 ± 0.754	1.579 ± 0.958
Dibenzo(a,e)pyrene	BDL	BDL	0.033 ± 0.031
Coronene	0.045 ± 0.036	BDL	0.513 ± 0.32

**Table 4:** Primary emission sources of carcinogenic metals and PAHs.

PM Species	Primary Emission Sources
As, Cd, and Ni	Industrial processes (e.g., metallurgical activities). Oil combustion.
Pb	Industrial activities, including the manufacturing of lead–acid batteries, iron and steel industries, and the production of ceramics. Non-tailpipe emissions.
Cr	Metal processing. Oil combustion. Vehicular emissions.
PAHs	Incomplete fuel combustion in vehicle exhausts and industrial stacks. Biomass burning.

**Table 5:** Comparison of total ${BaP}_{eq}$ values in this study with estimated values in the literature.

Study	Location	$\mathrm{Total}{BaP}_{eq}$ (ng/m^3^)
Current study	Los Angeles, US	0.1 ± 0.1
Current study	Thessaloniki, Greece	2.5 ± 1.78
Current study	Milan, Italy	3.5 ± 4.6
Wang et al. (2020) [98]	Wuhan, China	2.9 ± 1.4
Masiol et al. (2012) [99]	Venice, Italy	1.9 ± 2.6
Martellini et al. (2012) [73]	Florence, Italy	0.8
Kam et al. (2013) [97]	I-110 freeway in Los Angeles, US	12.7 ± 2.1
Kam et al. (2013) [97]	I-710 freeway in Los Angeles, US	23.3 ± 4.4
Kam et al. (2013) [97]	Surface streets in Los Angeles, US	8.6 ± 1.5

**Table 6:** Carcinogenic risks (×10^−6^) by inhalation of selected PM-bound toxic components for population in Los Angeles, Thessaloniki, and Milan. WS, BS, and MS indicate the Worst-case, Best-case, and Mixed scenarios, respectively. The values in bold represent the carcinogenic risk exceeding the US EPA standard.

	Los Angeles			Thessaloniki			Milan		
Species	WS	BS	MS	WS	BS	MS	WS	BS	MS
As	0.33 ± 0.10	0.21 ± 0.07	0.27 ± 0.09	**1.57 ± 0.78**	0.97 ± 0.48	**1.27 ± 0.63**	**2.08 ± 0.44**	**1.29 ± 0.27**	**1.69 ± 0.35**
Cd	0.04 ± 0.01	0.03 ± 0.01	0.03 ± 0.01	0.34 ± 0.19	0.21 ± 0.12	0.28 ± 0.16	0.46 ± 0.03	0.29 ± 0.02	0.38 ± 0.03
Cr(VI)	**2.69 ± 0.19**	**1.66 ± 0.12**	**2.17 ± 0.16**	**2.91 ± 1.01**	**1.80 ± 0.63**	**2.36 ± 0.82**	**9.82 ± 0.58**	**6.08 ± 0.36**	**7.95 ± 0.47**
Ni	0.22 ± 0.03	0.14 ± 0.02	0.18 ± 0.02	0.38 ± 0.23	0.23 ± 0.15	0.31 ± 0.19	0.6 ± 0.05	0.38 ± 0.03	0.49 ± 0.04
Pb	0.02 ± 0.01	0.01 ± 0.01	0.01 ± 0.01	0.06 ± 0.03	0.04 ± 0.02	0.05 ± 0.02	0.19 ± 0.02	0.12 ± 0.01	0.16 ± 0.02
${BaP}_{eq}$	0.06 ± 0.05	0.04 ± 0.04	0.05 ± 0.05	**1.25 ± 0.9**	0.77 ± 0.56	**1.01 ± 0.73**	**1.77 ± 2.33**	**1.1 ± 1.45**	**1.44 ± 1.89**
**Total**	**3.36 ± 0.39**	**2.07 ± 0.27**	**2.71 ± 0.34**	**6.51 ± 3.14**	**4.02 ± 1.96**	**5.28 ± 2.55**	**14.92 ± 3.45**	**9.26 ± 2.14**	**12.11 ± 2.80**

## Data Availability

The data that support the findings of this study are available on request from the corresponding author.

## Supplementary Materials

The following supporting information can be downloaded at: https://www.mdpi.com/article/10.3390/toxics11080697/s1, Figure S1: Comparison of (a) PAH and (b) carcinogenic metal concentrations (ng/m^3^) between the current study and Karageorgou et al. (2021) [75] and Besis et al. (2023) [76] studies. The concentration of Cr(VI) was obtained as 1/7 of the total Cr concentration.
