# Supplementary material for: Assessing Lifetime Cancer Risk Associated with Population Exposure to PM-Bound PAHs and Carcinogenic Metals in Three Mid-Latitude Metropolitan Cities
Source: Toxics. 2023 Aug 12;11(8):697. doi: 10.3390/toxics11080697 (PMC10457896; doi:10.3390/toxics11080697)
Supplement: Supplementary file 1 [file toxics-11-00697-s001.zip › toxics-2540974-SI.pdf]

toxics

Supplementary Materials: Assessing Lifetime Cancer Risk Associated with Population Exposure to PM-Bound PAHs and Carcinogenic Metals in Three Mid-Latitude Metropolitan Cities

Mohammad Aldekheel [1,2], Vahid Jalali Farahani [1] and Constantinos Sioutas [1,*]

[1] Department of Civil and Environmental Engineering, University of Southern California, Los Angeles, CA 90089, USA; aldekhee@usc.edu; jalalifa@usc.edu
[2] Department of Civil Engineering, Kuwait University, P.O. Box 5969, Safat 13060, Kuwait
* Correspondence: sioutas@usc.edu; Tel.: +1-213-740-6134; Fax: +1-213-744-1426

Supplementary information

(a)

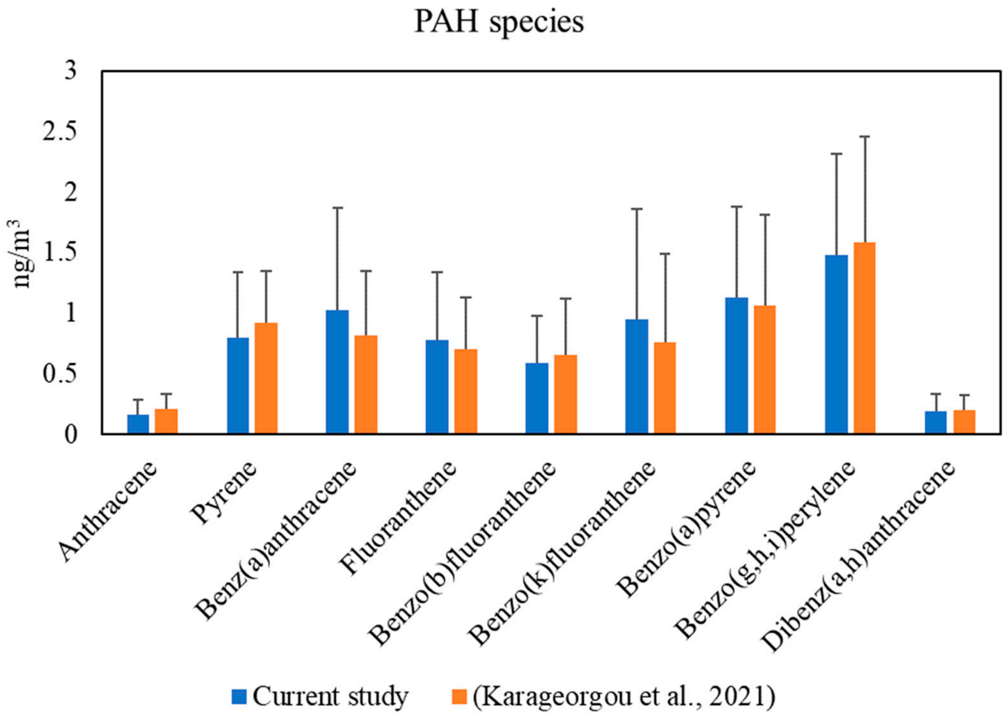

(b)

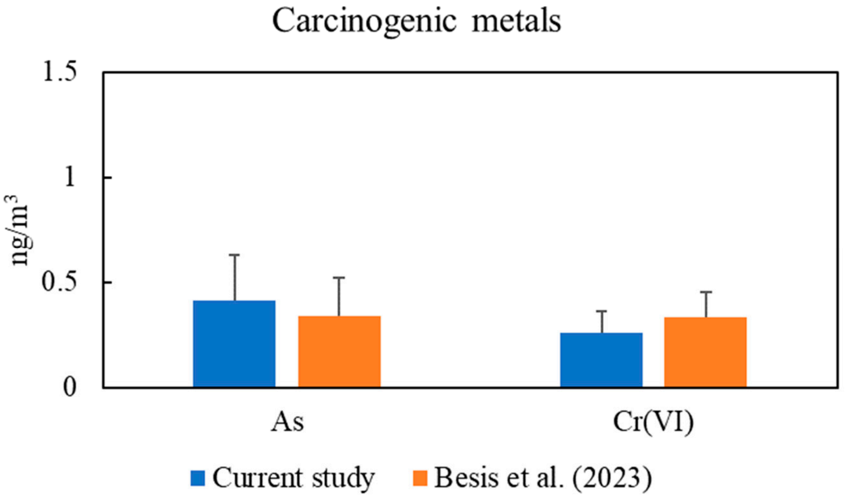

Figure S1. Comparison of (a) PAH and (b) carcinogenic metal concentrations (ng/m^3) between the current study and Karageorgou et al. (2021) [75] and Besis et al. (2023) [76] studies. The concentration of Cr(VI) was obtained as 1/7 of the total Cr concentration.
